# Supplementary material for: The Spatiotemporal Dynamics of Facial Movements Reveals the Left Side of a Posed Smile
Source: Biology (Basel). 2023 Aug 23;12(9):1160. doi: 10.3390/biology12091160 (PMC10525663; doi:10.3390/biology12091160)
Supplement: Supplementary file 1 [file biology-12-01160-s001.zip › biology-2530917-supplementary.pdf]

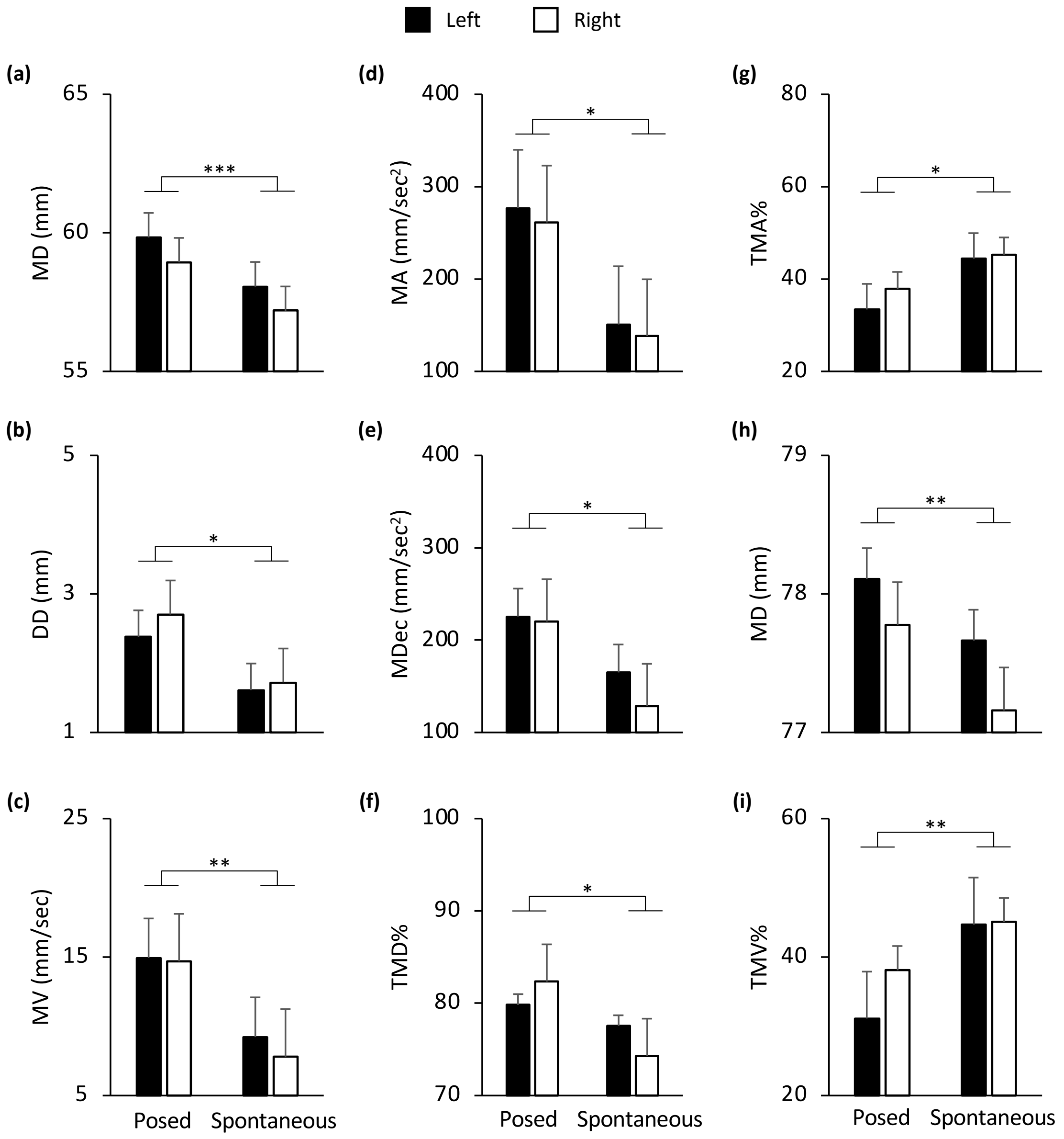

Figure S1. Experiment 1. Graphical representation of spatial, speed and temporal components of movement of the face during Posed and Spontaneous expressions of happiness. A significant main effect of Condition in the lower part of the face (i.e., cheilion markers, CH) was found for: (a) Maximum Distance (MD), (b) Delta Distance (DD), (c) Maximum Velocity (MV), (d) Maximum Acceleration (MA), (e) Maximum Deceleration (MDec), (f) Time to Maximum Distance (TMD%) and (g) Time to Maximum Acceleration (TMA%). A significant main effect of Condition in the upper part of the face (i.e., eyebrows, EB) was found for: (h) Maximum Distance (MD) and (i) Time to Maximum Velocity (TMV%). Error bars represent Standard Error. Asterisks indicate statistically significant comparisons (* = $p < 0.05$; ** = $p < 0.01$; *** = $p < 0.001$).

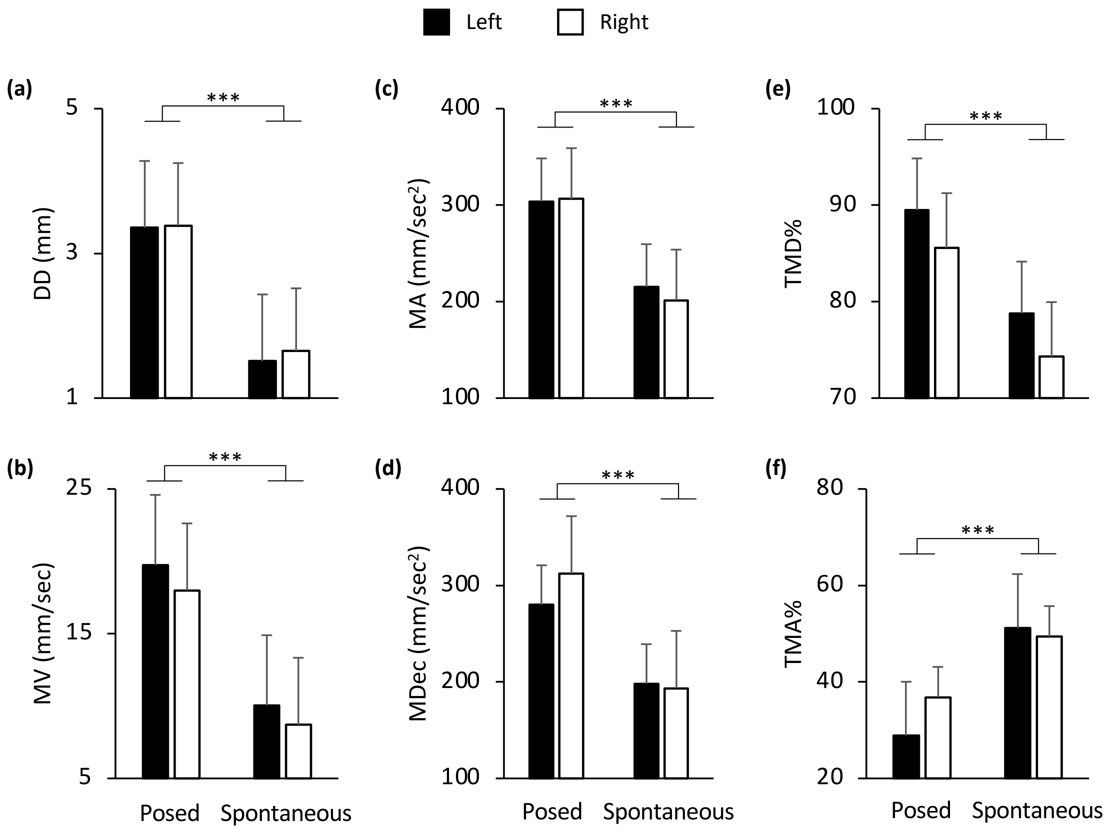

Figure S2. Experiment 2. Graphical representation of spatial, speed and temporal components of movement of the face during Posed and Spontaneous expressions of happiness. A significant main effect of Condition in the lower part of the face (i.e., cheilion markers, CH) was found for: (a) Delta Distance (DD), (b) Maximum Velocity (MV), (c) Maximum Acceleration (MA), (d) Maximum Deceleration (MDec), (e) Time to Maximum Distance (TMD%) and (f) Time to Maximum Acceleration (TMA%). Error bars represent Standard Error. Asterisks indicate statistically significant comparisons (*** = $p < 0.001$).

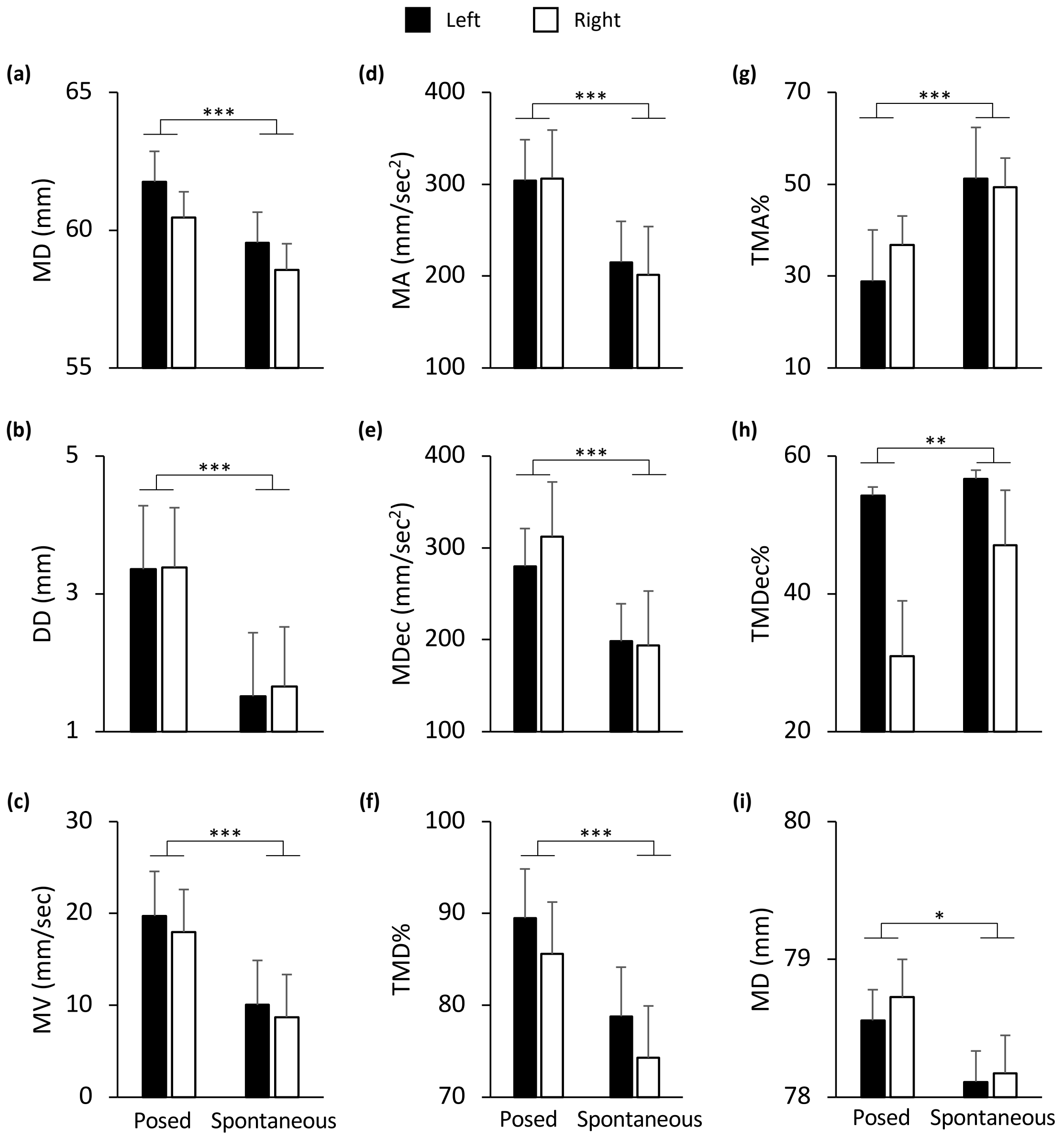

Figure S3. Mixed ANOVA (comparison analysis). Graphical representation of spatial, speed and temporal components of movement of the face during Posed and Spontaneous expressions of happiness. A significant main effect of Condition in the lower part of the face (i.e., cheilion markers, CH) was found for: (a) Maximum Distance (MD), (b) Delta Distance (DD), (c) Maximum Velocity (MV), (d) Maximum Acceleration (MA), (e) Maximum Deceleration (MDec), (f) Time to Maximum Distance (TMD%), (g) Time to Maximum Acceleration (TMA%), and (i) Time to Maximum Deceleration (TMDec%). A significant main effect of Condition in the upper part of the face (i.e., eyebrows, EB) was found for (i) Maximum Distance (MD). Error bars represent Standard Error. Asterisks indicate statistically significant comparisons (* = $p < 0.05$; ** = $p < 0.01$; *** = $p < 0.001$).
